# Compromised Vision Impairs Successful Aging Among Assisted Living Residents

**DOI:** 10.1093/geroni/igaa057.1708

**Published:** 2020-12-16

**Authors:** Judith Scott, Ann Mayo

**Affiliations:** 1 University of Colorado Colorado Springs, Colorado Springs, Colorado, United States; 2 University of San Diego, San Diego, California, United States

## Abstract

Community dwelling independent older adults’ successful aging is known to be hampered by sensory and cognitive impairments. However, little is known about to what degree these impairments affect successful aging among assisted living (AL) residents. The purpose of this quantitative study, conducted in three (AL) facilities, was to describe factors affecting successful aging. A total of 88 older adults (M=89.0, SD=7.54), mostly women (n=68), completed hearing (CALFRAST-Strong procedure at 75cm, 35cm, and 2cm), vision (Jaeger reading [proximate], Snellen Acuity [visual acuity]), and cognitive screening (MiniCog, Borson et al), as well as the Lawton Instrumental Activities of Daily Living (IADL) and Successful Aging Inventory (SAI, Troutman et al, 2011). Most (68%) demonstrated hearing loss >25DB, with a significant difference demonstrated between age groups (age 65-89; n=38) (90-100; n=49) with the older group demonstrating worse hearing (F(1,80)=5.9, p=.017). Some vision compromise was noted for both reading (14.3%) and visual acuity (10.8%). Over one third of participants (34.1%) demonstrated compromised cognition. The SAI results indicated most participants were managing IADLs well (M=6.11, SD=1.42) and aging successfully (M=63.39, SD=9.04). Hearing, cognition and IADLs were not significantly related to successful aging. However, when compared to those without vision issues, participants with compromised vision, both reading and visual acuity, scored significantly lower on the SAI (reading F(1,75)= 24.9,p=.000; visual acuity F(1,28)=4.31, p=000). The infrastructure provided by AL settings may compensate for hearing, cognition, and IADL problems, but not as well for vision problems. Interventions supporting AL residents’ vision should be a priority to improve successful aging.

